# Supplementary figures and images for: Large-scale, dynamin-like motions of the human guanylate binding protein 1 revealed by multi-resolution simulations
Source: PLoS Comput Biol. 2019 Oct 7;15(10):e1007193. doi: 10.1371/journal.pcbi.1007193 (PMC6797221; doi:10.1371/journal.pcbi.1007193)

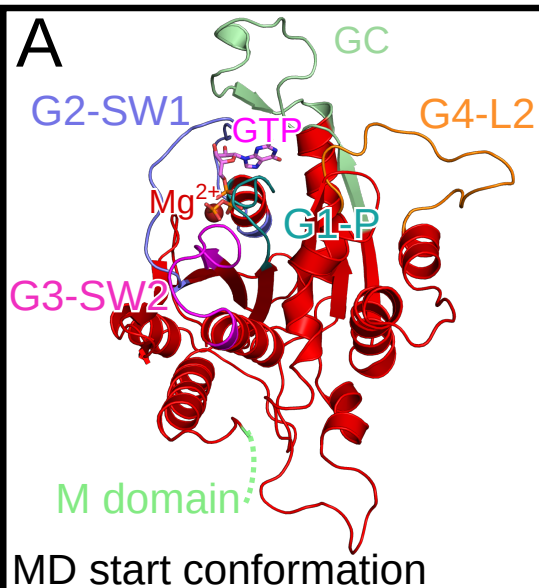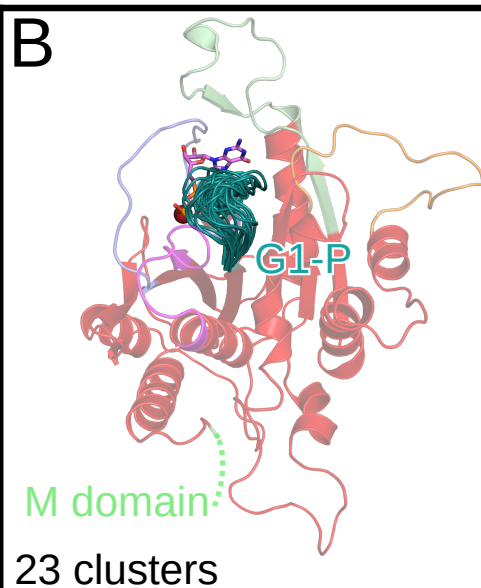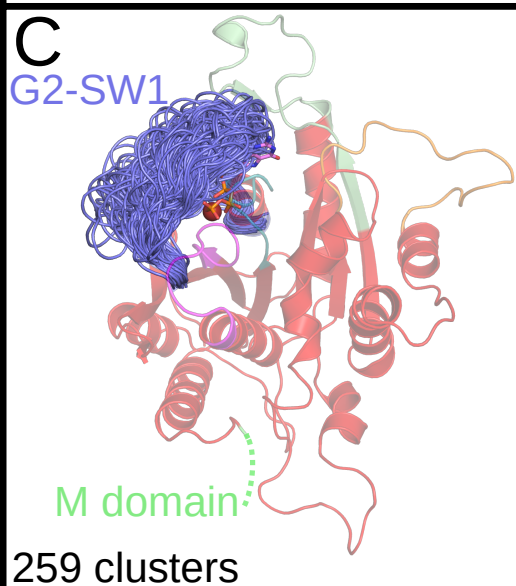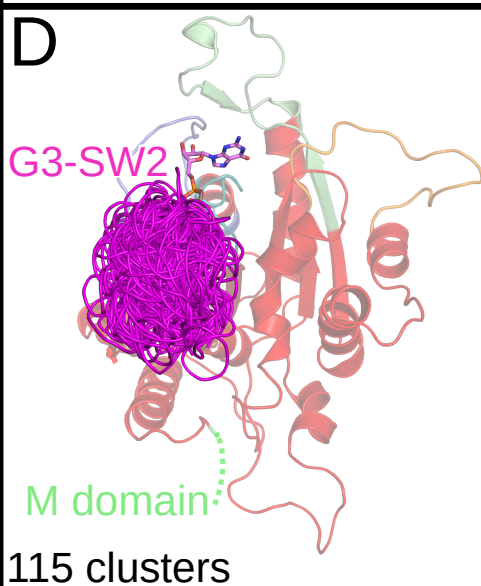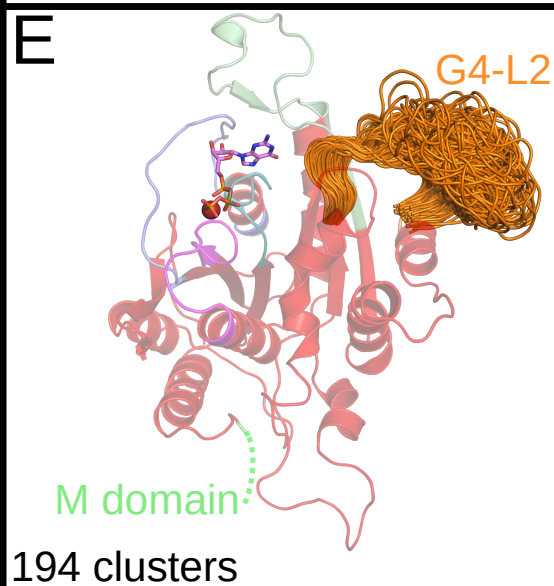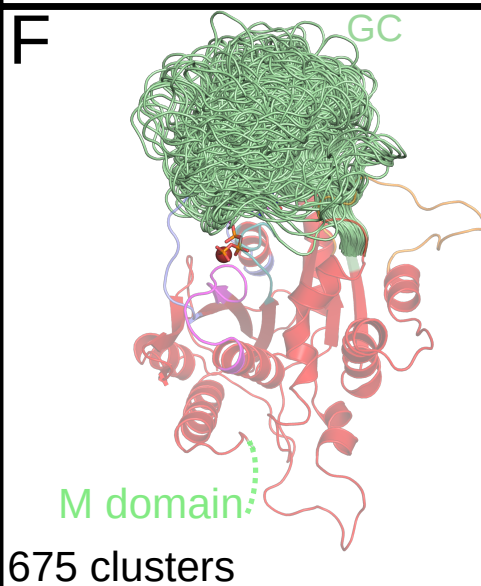

Supplement: S1 Fig — (A) Initial conformation of the LG domain from which the H-REMD simulation of the hGBP1 monomer was started. The central conformations for all clusters obtained for (B) G1-P, (C) G2-SW1, (D) G3-SW2, (E) G4-L2, and (F) GC are shown. The number of clusters for each loop are given, too. The coloring is the same as in Fig 3. For clarity, GTP (violet) and Mg2+ (carmine) are also shown, allowing to distinguish open and closed loop conformations from each other. However, it is emphasized that GTP and Mg2+ were not present in the H-REMD simulation of the hGBP1 monomer. (PDF) [file pcbi.1007193.s001.pdf]

**A**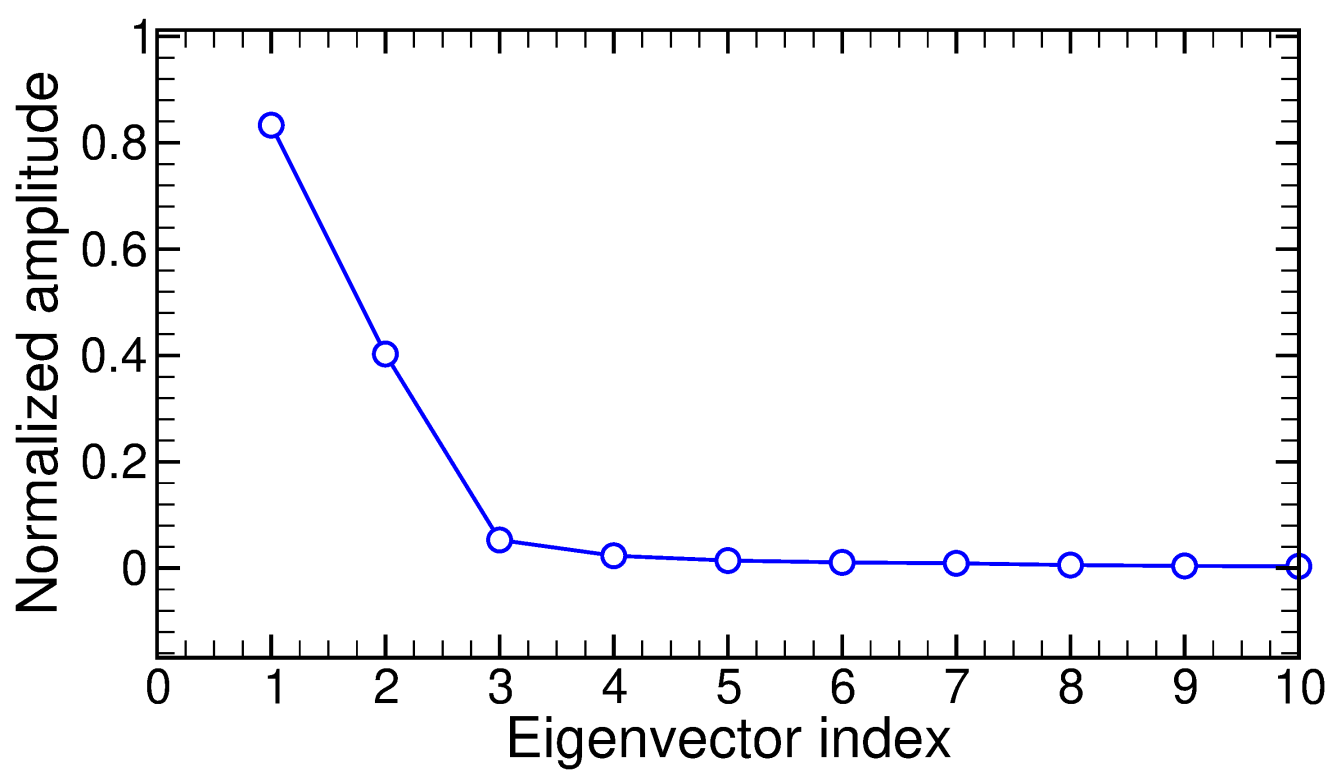**B**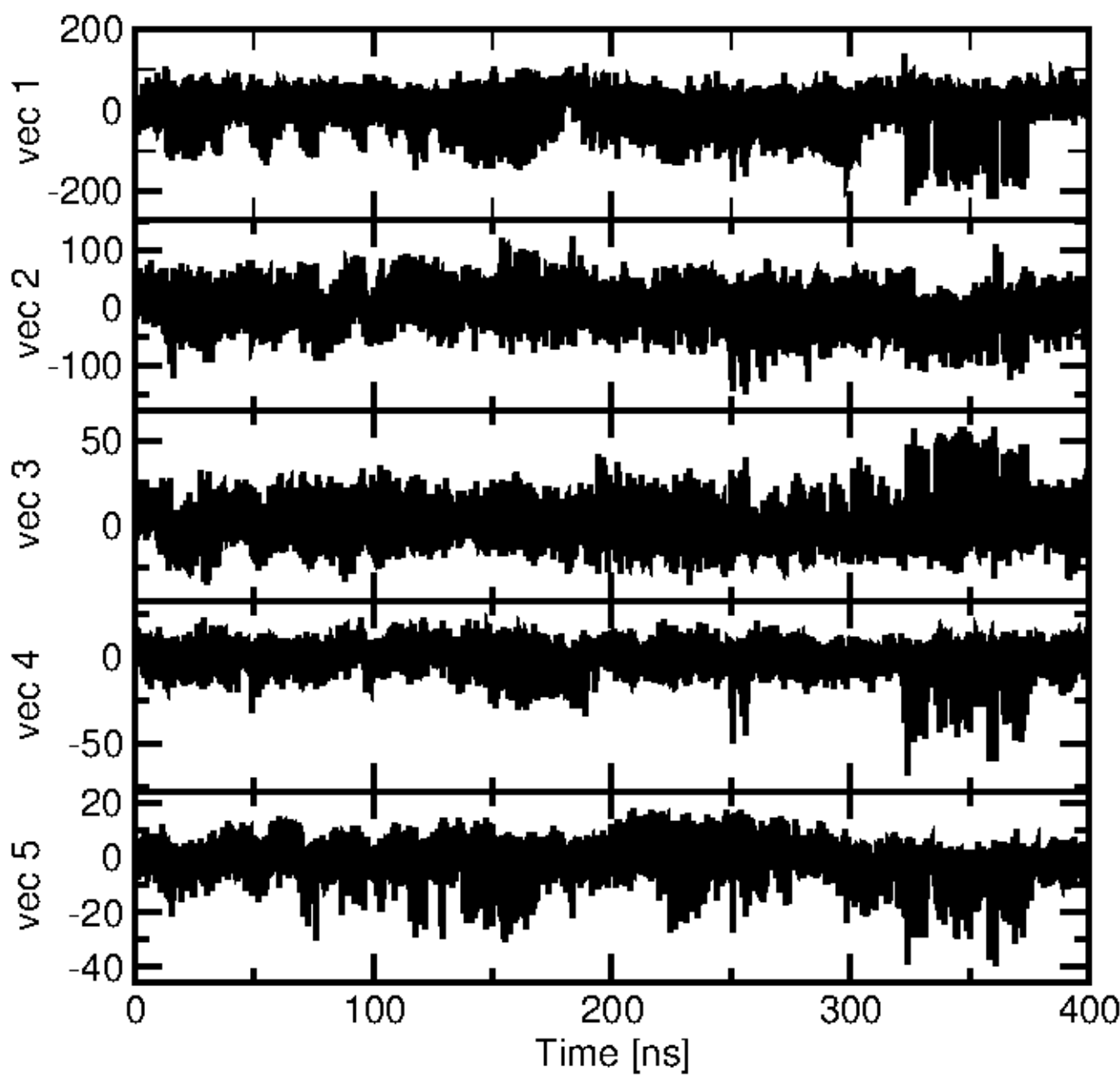

Supplement: S2 Fig — (A) Amplitude of the first ten eigenvalues obtained from principal component analysis (PCA) of the all-atom H-REMD simulation of the hGBP1 monomer. (B) Projection of the unbiased H-REMD replica onto the first five PCA eigenvectors. (PDF) [file pcbi.1007193.s002.pdf]

A

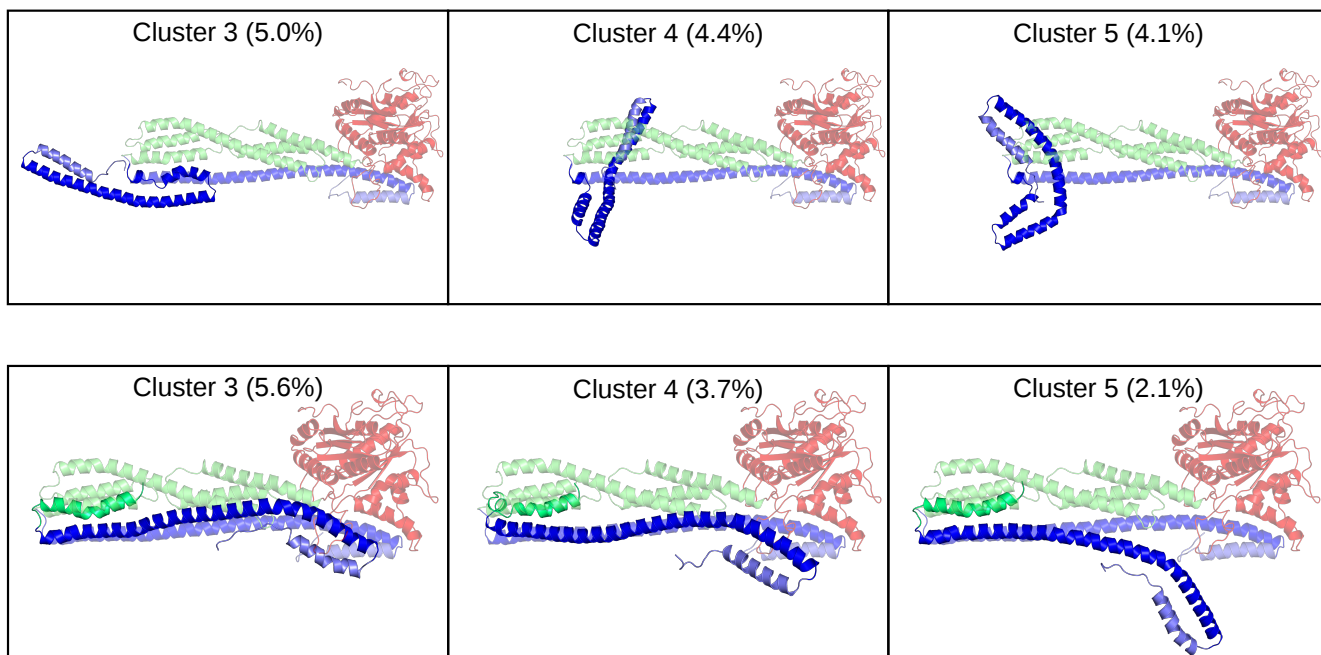

B

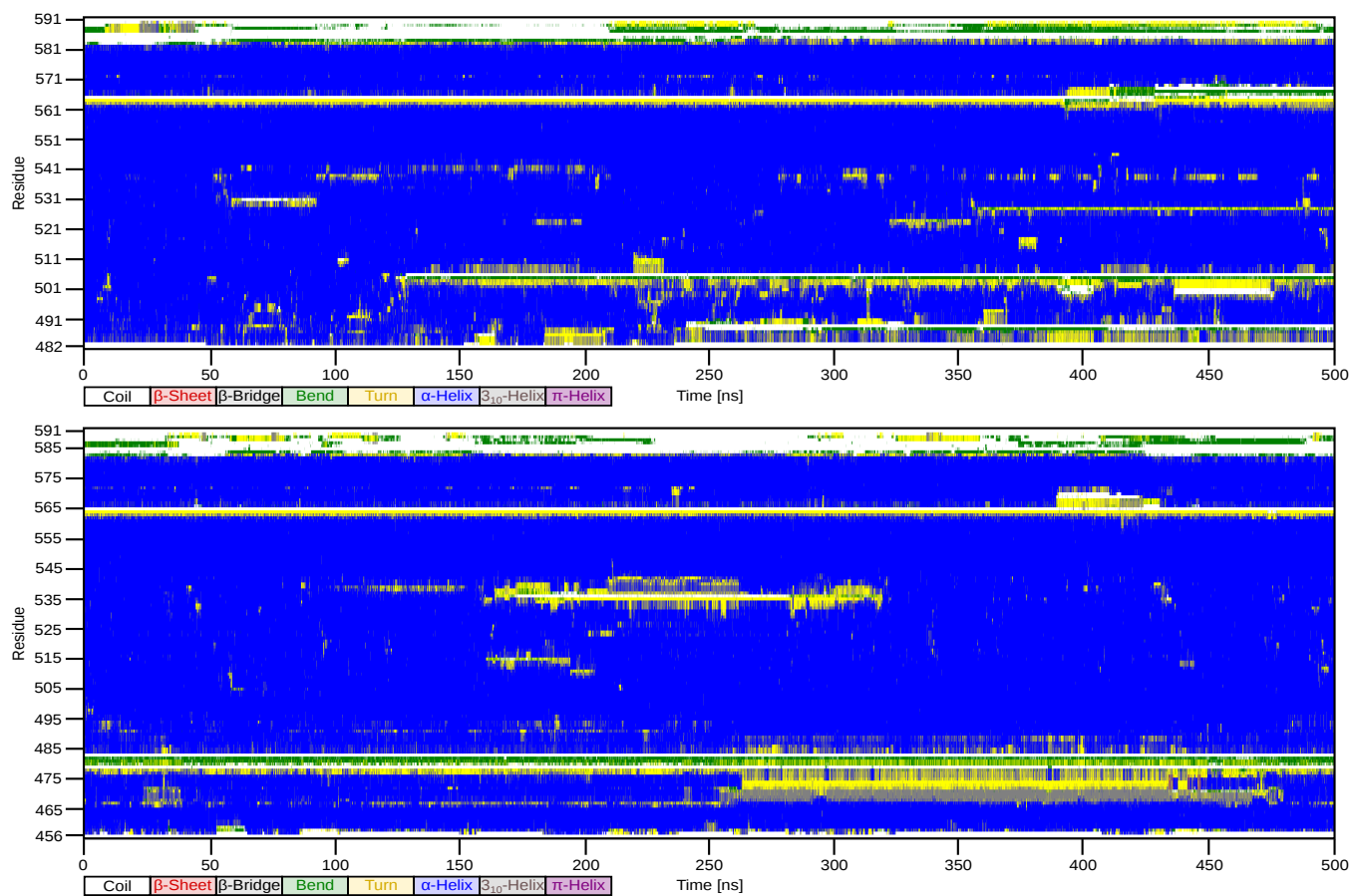

Supplement: S3 Fig — (A) Representative conformations for clusters 3–5 with their occurrence (in %) obtained from the MD simulations of the isolated E domain (top) and of helix α11 plus the E domain (bottom). The cluster structures were aligned to the crystal structure of full-length hGBP1 (shown as transparent cartoon) using residues 482–484 for the alignment of the isolated E domain and α11 for alignment of α11 plus the E domain. (B) Evolution of the secondary structure shown for each residue as a function of time for the isolated E domain (top) and for helix α11 plus E domain (bottom). (PDF) [file pcbi.1007193.s003.pdf]

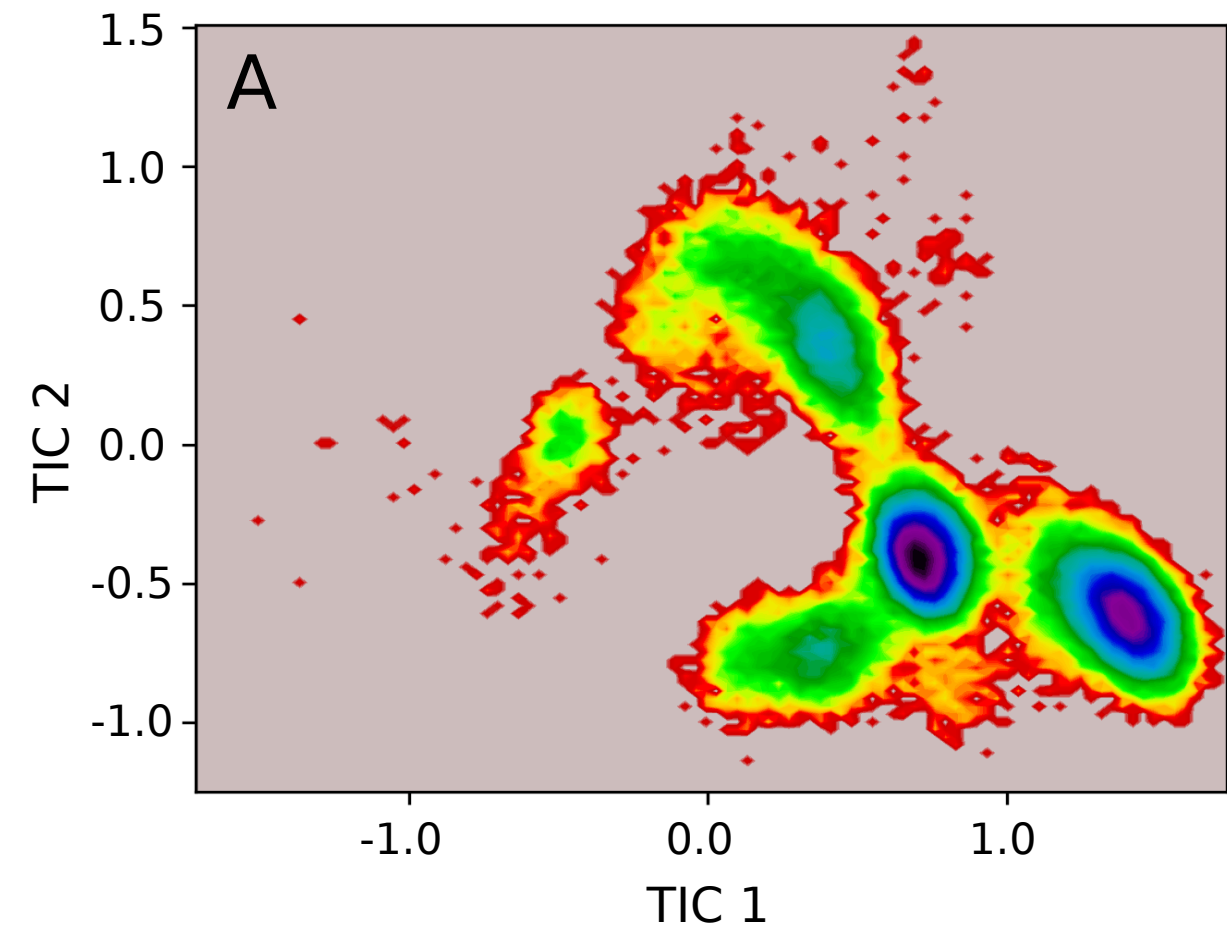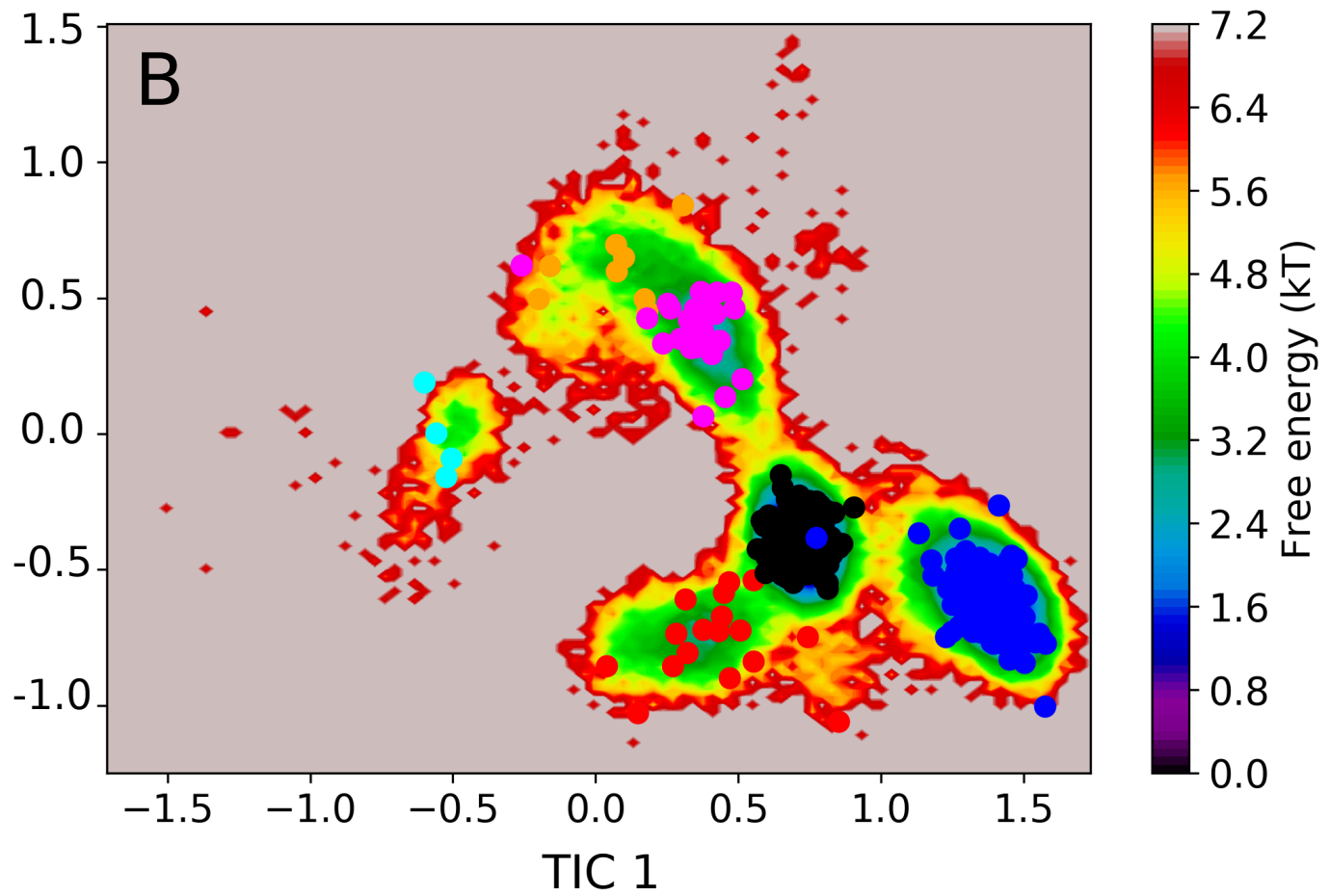

Supplement: S4 Fig — (A) Free energy surface plotted along the first two eigenvectors obtained from TICA. The free energy values correspond to the color scale on the right (in kB T as the Boltzmann constant and T = 310 K). (B) Fuzzy PCCA+ clustering of the microstates resulted in six macrostates. The microstates are projected onto the first two TICs and their membership to one of the six macrostates is identified by different colors. (PDF) [file pcbi.1007193.s004.pdf]

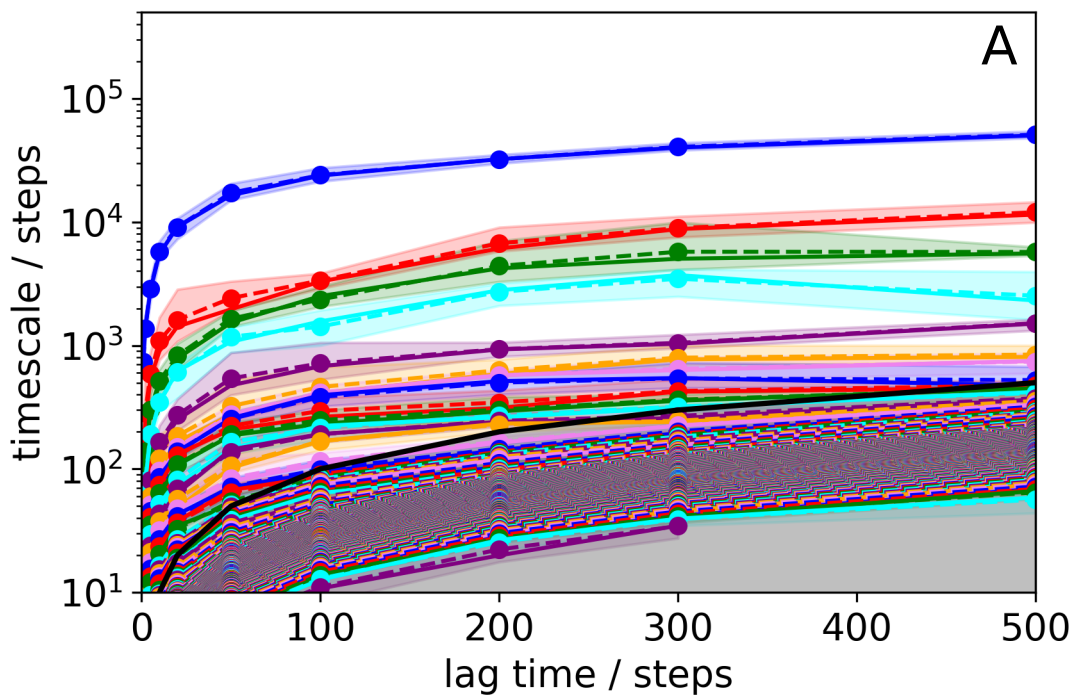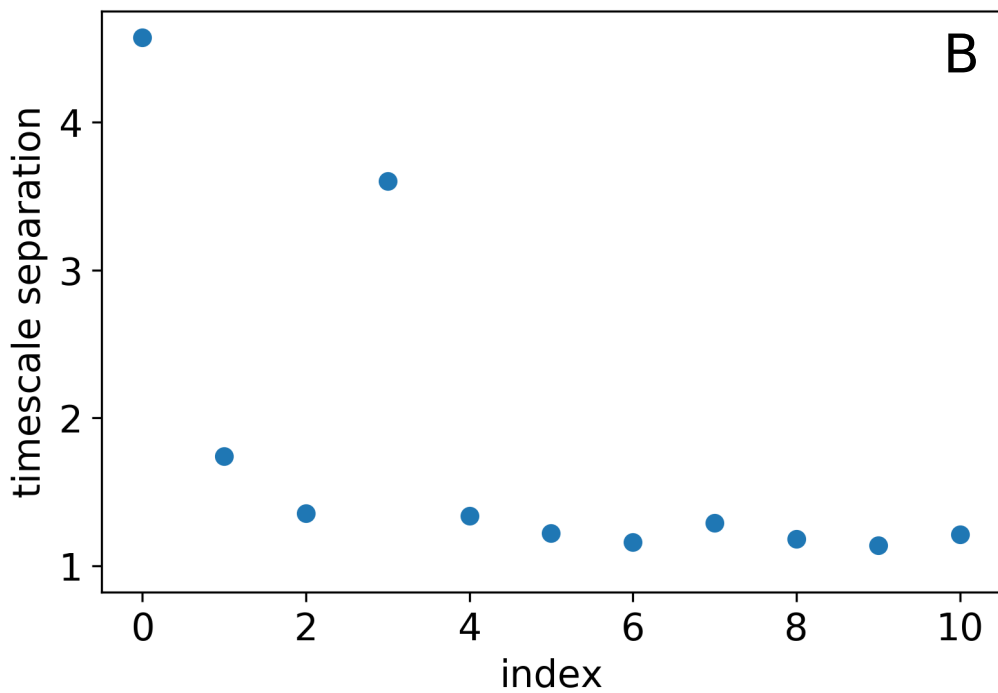

Supplement: S5 Fig — (A) Convergences of the implied timescales derived from MSMs for different lag times. One step corresponds to 1.5 ns. (B) Relative implied time scales for a lag time of 450 ns. (PDF) [file pcbi.1007193.s005.pdf]

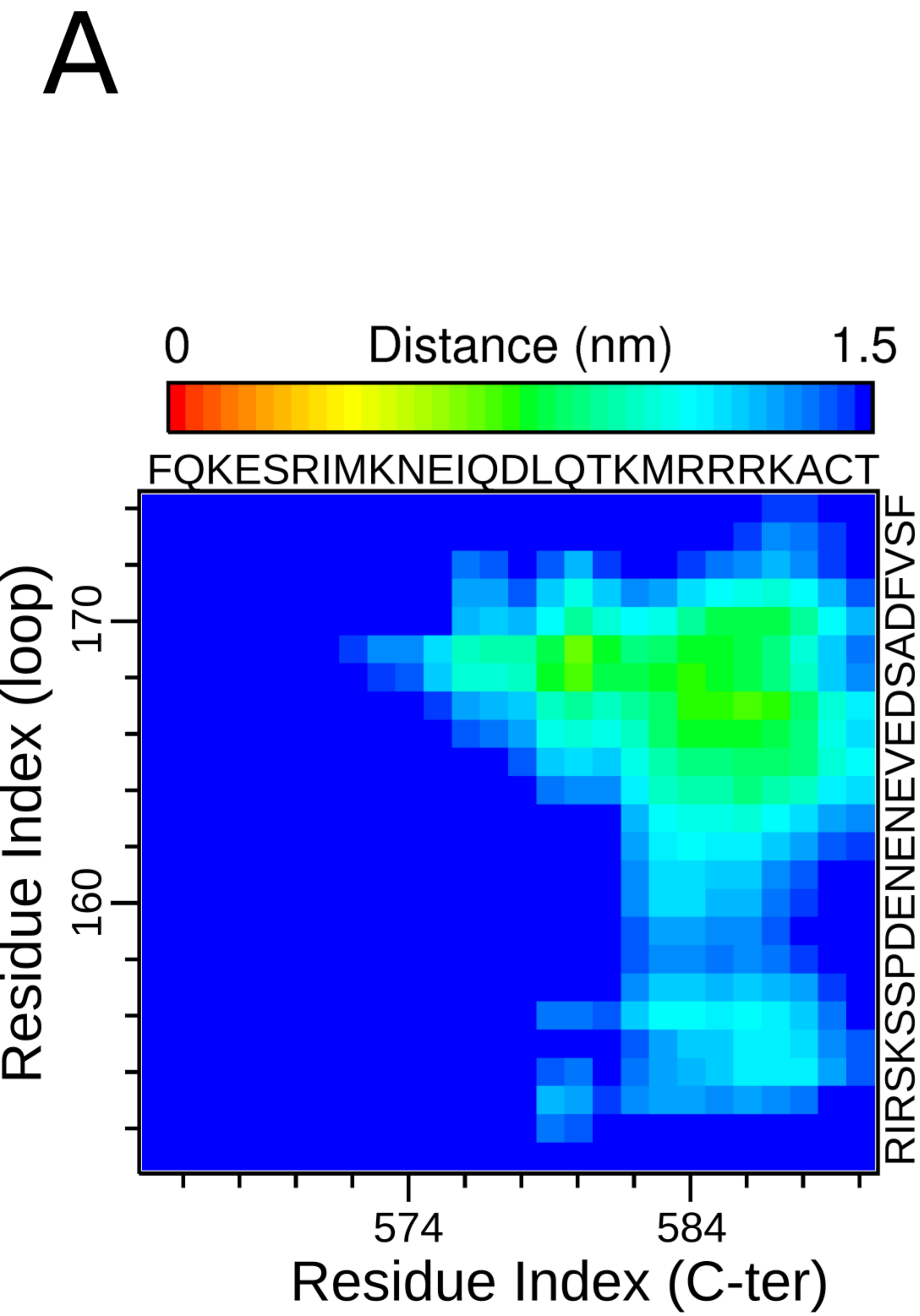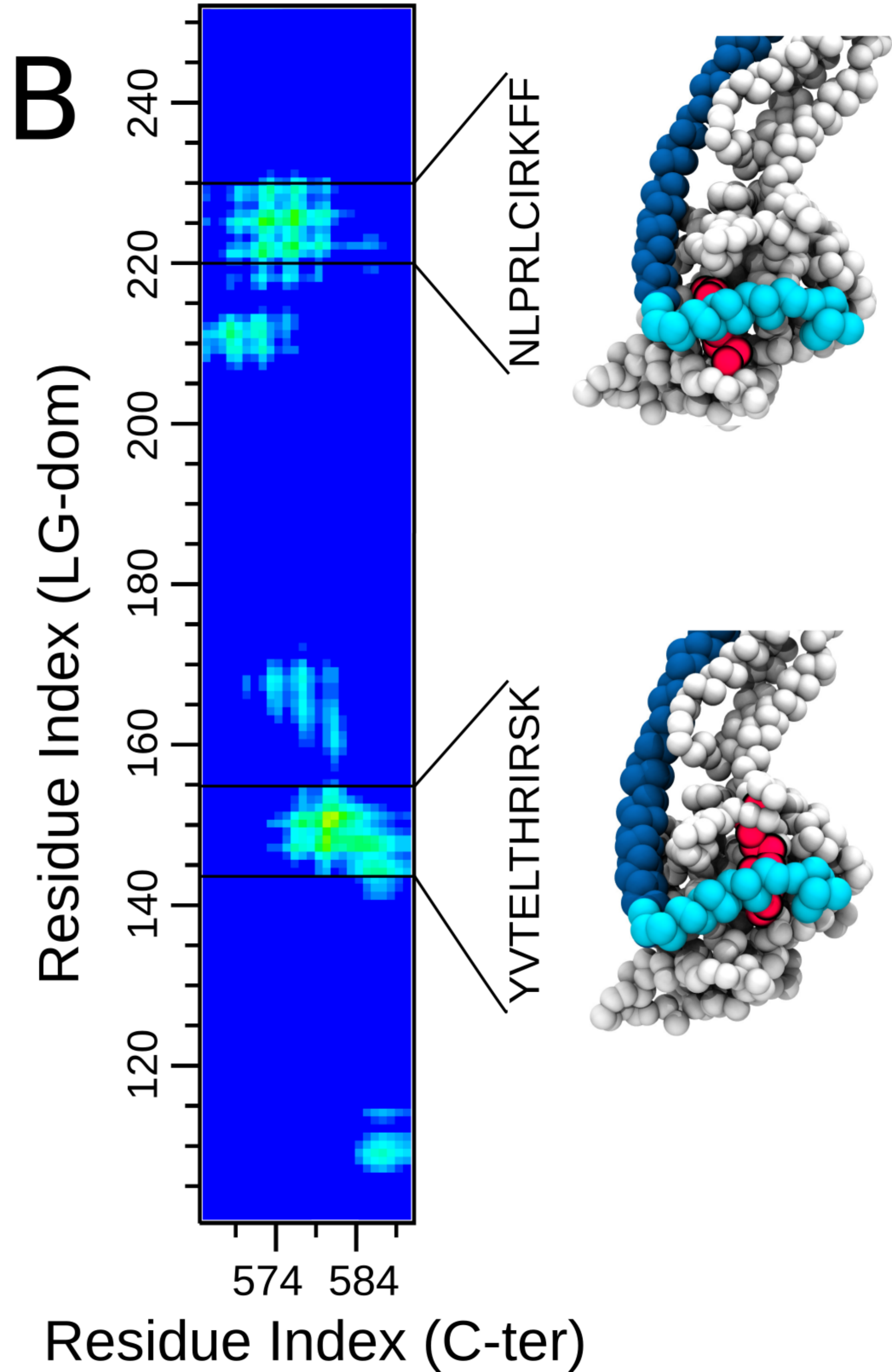

Supplement: S6 Fig — (A) Distance map between the Cα atoms of the C-terminal region, including the helix α13, and the loop formed by residues R151–F174 during transition from the Markov state A to other states in the MSM. The residue index on the x and y axes is accompanied by the corresponding amino acid sequence on the respective opposite side of the plot. (B) Cα-distance map between the C-terminal region as in (A) and relevant residues of the LG-domain calculated for conformations belonging to the Markov state 4. The sequences of the LG domain stretches that form strong contacts with the C-terminal region are displayed on the right, together with structural snapshots where the same residues are highlighted in red, while the helices α12 and α13 are shown on blue and cyan, respectively. (PDF) [file pcbi.1007193.s006.pdf]

A

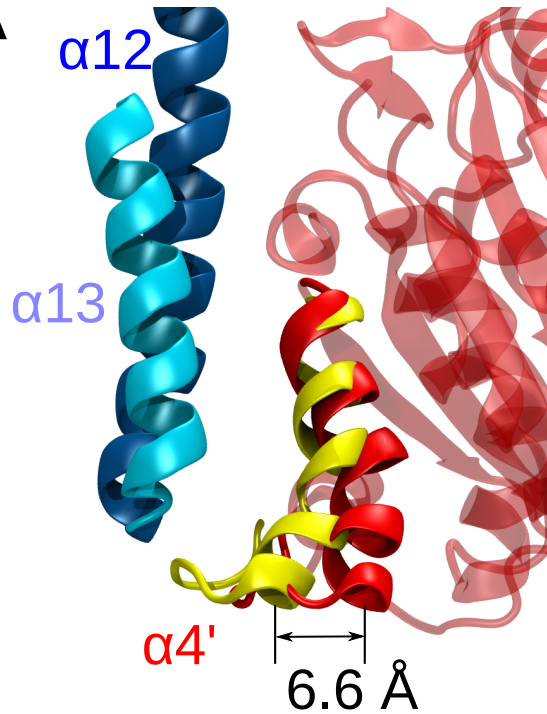

B

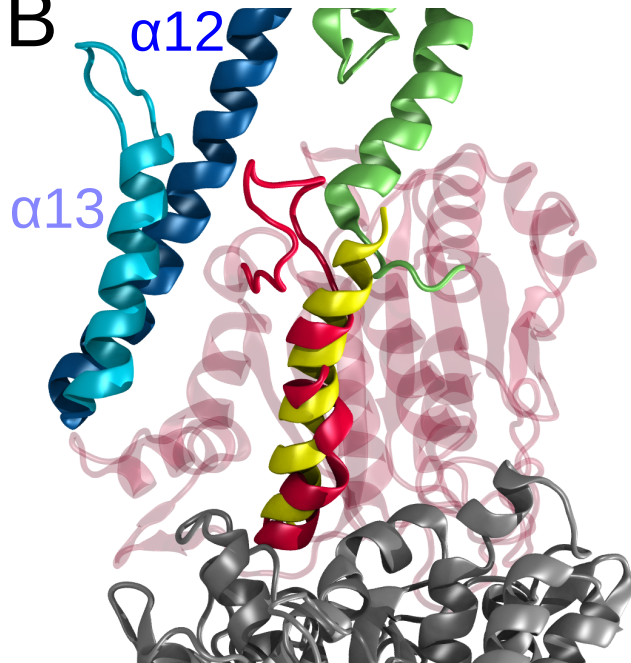

Supplement: S7 Fig — (A) Comparison of the position of helix α4’ from the hGBP1 monomer (PDB 1DG3, red) with that in the LG domain dimer (PDB 2B92, yellow). This is the major conformational difference between the LG domain monomer and dimer. (B) Comparison of the position of helix α3 in the LG domain dimer (PDB 2B92, yellow) with that from the hGBP1 monomer (PDB 1DG3, red). In order to avoid atom clashes when building the full-length hGBP1 dimer, the yellow helix had to be replaced with the red one. (PDF) [file pcbi.1007193.s007.pdf]

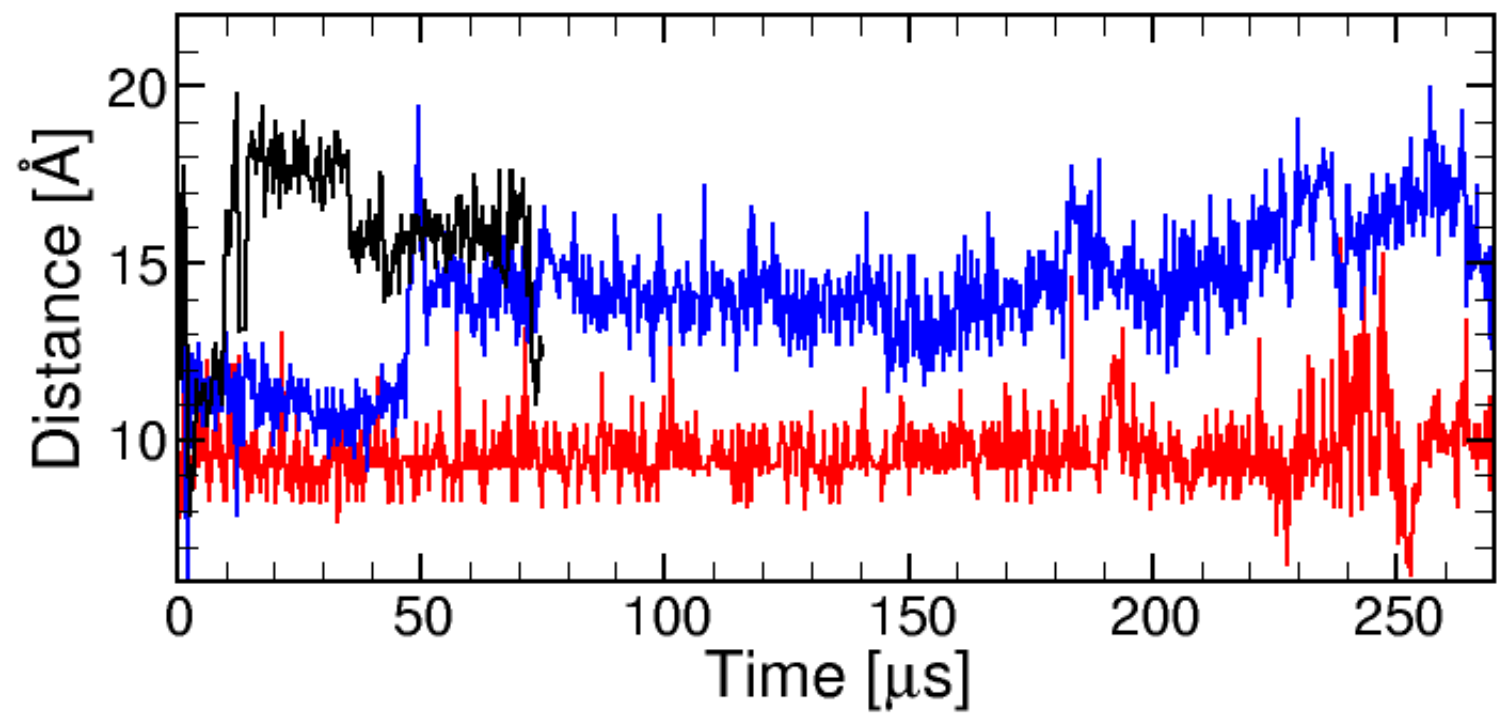

Supplement: S8 Fig — Distance between the side chains of E217 and K567 for representative Martini simulations of (black) the hGBP1 monomer at 310 K (obtained from the 63 μs simulation), (red) and (blue) the hGBP1 dimer at 320 K (simulated for 270 μs). In one of the monomers composing the dimer the helix α13 moves (blue) while it does not in the other monomer (red). This motion correlates to the presence of the salt bridge between E217 and K567. In the monomer this salt bridge is never formed (black) as these two residues are too far away from each other. In the dimer, helix α4’ adopts a slightly different position than in the monomer (see S7 Fig), allowing a salt bridge being formed between E217 and K567. This salt bridge impedes the motion of α13 in the dimer. Only if the temperature is raised to 320 K, α13 starts moving in one of the monomers composing the dimer (blue) while it remains intact in the other one (red). At 50 μs the salt bridge in the monomer with the flexible α13 is completely broken, which corresponds to the time when this helix has adopted the 90° rotated position (see Fig 7). It should be noted that the distances shown here are between the centers of the coarse-grained side-chain beads and are therefore larger than the atom-based distances usually reported for salt bridges. (PDF) [file pcbi.1007193.s008.pdf]

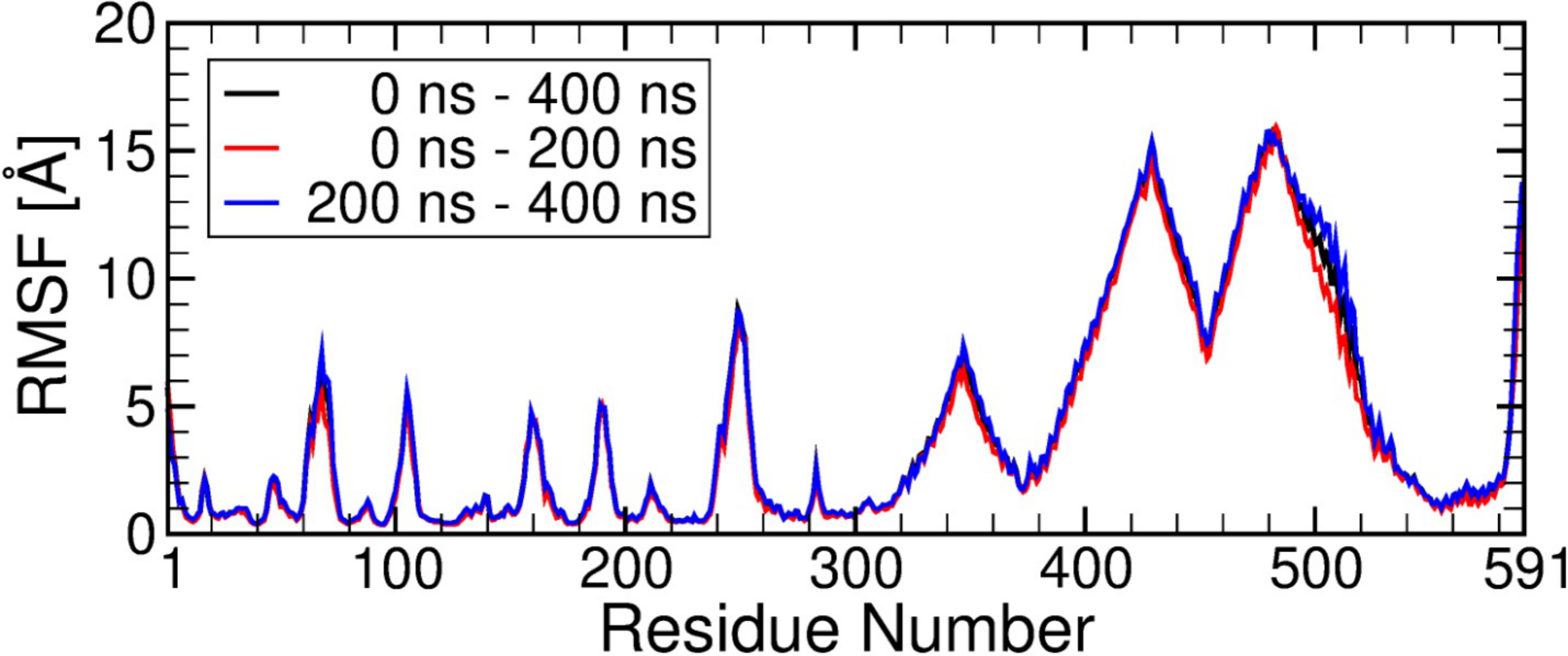

Supplement: S10 Fig — The fluctuations of hGBP1 during the first half of the H-REMD simulation (red), the second half (blue) and the full simulation (black) are almost identical, showing that no new conformations are sampled in the second half of the simulation. The results are shown as RMSF values calculated for the target replica (310 K, no energy bias). (PDF) [file pcbi.1007193.s010.pdf]
