# Supplementary material for: Large-scale, dynamin-like motions of the human guanylate binding protein 1 revealed by multi-resolution simulations
Source: PLoS Comput Biol. 2019 Oct 7;15(10):e1007193. doi: 10.1371/journal.pcbi.1007193 (PMC6797221; doi:10.1371/journal.pcbi.1007193)
Supplement: S9 Fig — The final snapshot of the dimer obtained from the coarse-grained MD simulation at 320 K was backmapped to the all-atom level. Hence this dimer is called CG-to-AA dimer in the following. The initial CG-to-AA dimer structure is shown in (A) and compared to the dimer model produced for starting the Martini simulations (gray) in (B). Snapshots of the CG-to-AA dimer obtained at 50 and 100 ns of the all-atom simulation are displayed in (C) and (D). The RMSF calculated from that trajectory is projected onto the dimer structure in (E) according to the color scale given in that panel. The evolution of the Q577–Q577 distance during the simulation is plotted in (F). In (G) the conformational clusters of the loops of both LG domains are displayed. The same coloring scheme for hGBP1 as used in the other figures is employed. GTP and Mg2+, which were present in this simulation, are shown in magenta and carmine, respectively. (PDF) [file pcbi.1007193.s009.pdf]

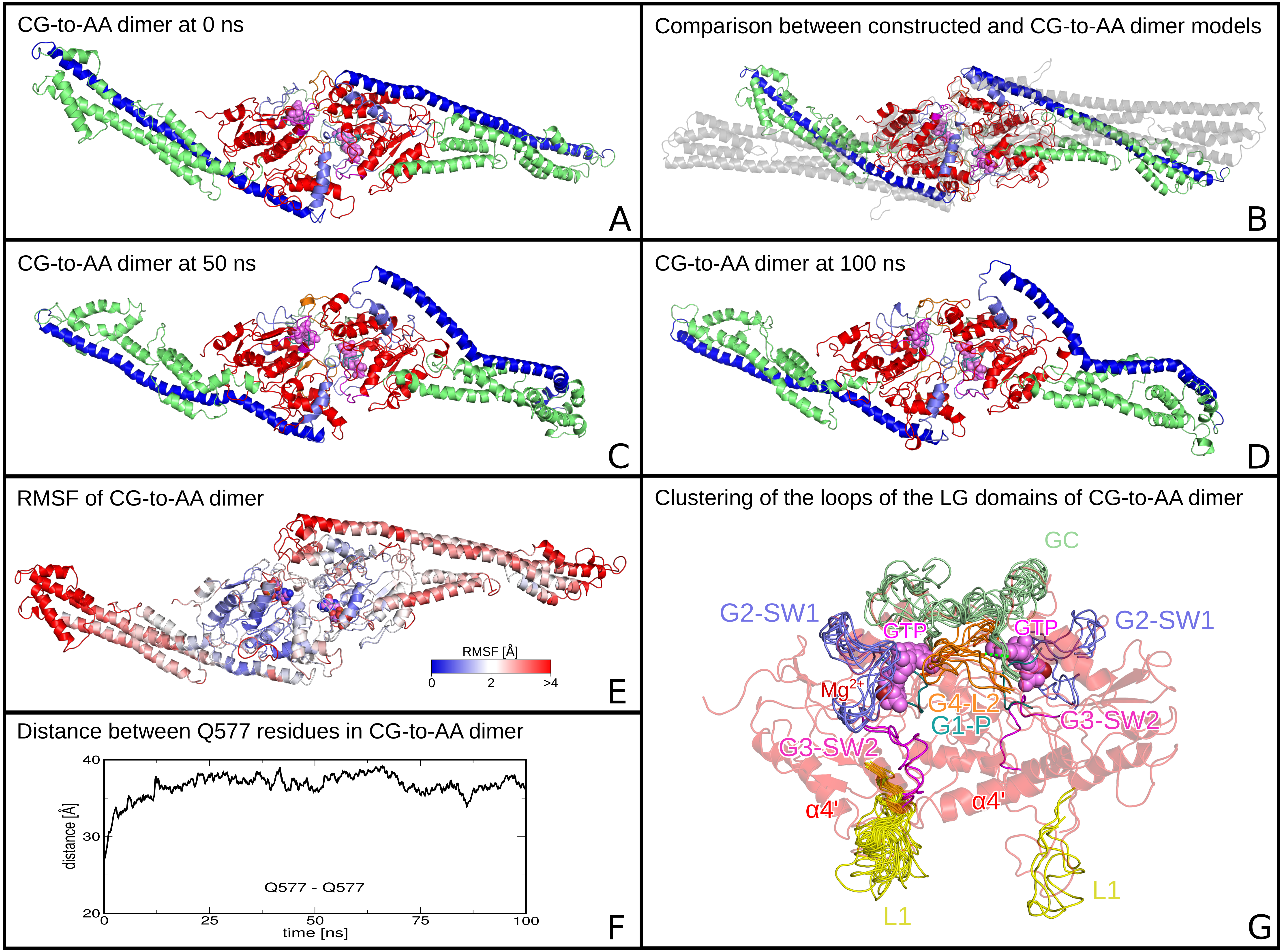
CG-to-AA dimer at 0 ns
A
Comparison between constructed and CG-to-AA dimer models
B
CG-to-AA dimer at 50 ns
C
CG-to-AA dimer at 100 ns
D
RMSF of CG-to-AA dimer
RMSF [Å]
0
2
>4
E
Distance between Q577 residues in CG-to-AA dimer
40
30
20
distance [Å]
Q577 - Q577
0
25
50
75
100
time [ns]
F
Clustering of the loops of the LG domains of CG-to-AA dimer
GC
G2-SW1
G2-SW1
GTP
GTP
Mg2+
G4-L2
G1-P
G3-SW2
G3-SW2
α4'
α4'
L1
L1
G
