# Supplementary material for: Large-scale, dynamin-like motions of the human guanylate binding protein 1 revealed by multi-resolution simulations
Source: PLoS Comput Biol. 2019 Oct 7;15(10):e1007193. doi: 10.1371/journal.pcbi.1007193 (PMC6797221; doi:10.1371/journal.pcbi.1007193)
Supplement: S1 Table — (PDF) [file pcbi.1007193.s011.pdf]

## Supporting Information - Table

| RESOURCE | SOURCE | IDENTIFIER |
|---|---|---|
| | | |
| **Software and Algorithms** | | |
| ModLoop | (49, 50) | https://modbase.compbio.ucsf.edu/modloop/ |
| Gromacs 2016, 4.5.5 | (52, 54, 55) | http://www.gromacs.org/ |
| PLUMED version 2.0 | (53) | https://www.plumed.org/ |
| Maestro | (60) | https://www.schrodinger.com/maestro |
| Gaussian 09 | (66) | https://gaussian.com/ |
| VMD | (68) | https://www.ks.uiuc.edu/Research/vmd/ |
| PyMol | (69) | https://pymol.org/ |
| DSSP | (70) | https://swift.cmbi.umcn.nl/gv/dssp/ |
| PyEMMA | (73) | http://emma-project.org/ |
| | | |
| **Databases** | | |
| PDB files | Protein Data Bank | https://www.rcsb.org/ |
