# Supplementary material for: Large-scale, dynamin-like motions of the human guanylate binding protein 1 revealed by multi-resolution simulations
Source: PLoS Comput Biol. 2019 Oct 7;15(10):e1007193. doi: 10.1371/journal.pcbi.1007193 (PMC6797221; doi:10.1371/journal.pcbi.1007193)
Supplement: S1 File — (PDF) [file pcbi.1007193.s012.pdf]

Supporting Information - README

A public Open Science Framework project account has been created: https://osf.io/a43z2/

In this project account, the data is provided allowing to repeat the simulations by providing the necessary GROMACS input files (.gro or .pdb, .tpr, .mdp) and to access the resulting trajectories (.xtc and .pdb for reading the .xtc files). The files can be found in following folders:

hGBP1 monomer HREMD

Contains the input data for the all-atom HREMD simulation and the trajectory for the target replica that was analyzed.

hGBP1 truncated models

Contains the data for the all-atom MD simulations of the E domain of hGBP1 and of helix α11 + E domain.

hGBP1 monomer Martini-MD

Contains the data for the five coarse-grained simulations of the hGBP1 monomer.

hGBP1 dimer Martini-MD

Contains the data for the six coarse-grained simulations of the hGBP1 dimer. One of these simulations was run at 320 K as indicated by the corresponding file names.

hGBP1 backmapped dimer all-atom MD

Contains the data for the all-atom MD simulation of the dimer backmapped from the coarse-grained to the all-atom level.

Running and analyzing the MD simulations

The use of GROMACS 2016 and 4.5.5, PLUMED version 2.0, and the different analysis tools invoked via GROMACS is described in Methods.

For the calculation of the Markov state models from the Martini-MD simulations of the hGBP1 dimer the Jupyter notebook used in conjunction with PyEMMA is provided (in the *hGBP1 monomer Martini-MD* folder).
